# Pain Management in Pediatric Age Group

**DOI:** 10.4103/0973-1075.53590

**Published:** 2009

**Authors:** R Namrata, PV Ramamani, G Saroja

**Affiliations:** Department of Anaesthesiology and Pain Relief, Kidwai Memorial Institute of Oncology, Bangalore, India

**Keywords:** Cancer pain, Oral morphine, Pediatric

## Abstract

The management of pain in palliative care of children is somewhat different from that in adults. The use of opioids in pediatric palliative care presents some unique challenges. Confident and rational use of opioids, illustrated by WHO Guidelines is essential for adequate management of pain in children with life limiting conditions.

## INTRODUCTION

Palliative care in children poses many challenges starting from assessment to counseling. Reference from paeditric oncology unit to palliative care is relatively less due to various reasons related to type of malignancy, incidence of pain, more so in children in the age group of below five years. Despite two decades of experience, we tend to go slow and cautious in titrating and escalating dose of morphine in children.

These two case reports demonstrate the need for being more aware of pain problems and palliative care challenges associated with children, especially below five years of age.

## CASE REPORTS

### Case 1

A 2-year-old girl weighing 9.5 kg was referred to department of palliative care in the month of September ‘08 with the diagnosis of neuroblastoma stage IV and disseminated systemic disease. The child presented with proptosis of right eye with periorbital ecchymosis and secondary deposits over scalp. She had developed paraparesis [secondary to lumbar mass]. There was no role for oncological intervention at the time of reference to palliative care. On presentation to palliative care department, child was crying incessantly, had no relief with ibuprofen syrup and was started on oral morphine solution 1 mg 8 hourly with nimesulide syrup 5 ml BD. After 24 hrs she had taken 4 prn doses besides three regular doses. The parents had to carry the child on the shoulders and walk around the ward round the clock. She could not be made to sleep on the bed. By 36 hrs she was titrated to receive 2 mg 4^th^ hrly along with nimesulide syrup. Constipation was managed with lactulose syrup 5 ml on alternate days. She had no other side effects. She was comfortable, sedated but arousable. Parents were counseled and had accepted the prognosis well. They wanted pain relief and comfort for the child. They were taught eye care and dressing for the scalp metastases. She was discharged after 72 hrs and was on regular follow up every fortnight till death in the second week of December.

The morphine requirement escalated as follows. On discharge 2 mg 4^th^ hrly with prn doses. On first visit it was increased to 5 mg 4^th^ hrly. At second follow up it was increased to 7.5 mg 4^th^ hrly. At the subsequent visit, it was increased to 10 mg 4^th^ hrly with prn doses till death. Parents returned the remaining oral morphine solution to the department after the death of the child and reported that child was comfortable, feeding, and sleeping well until death.

### Case 2

A 2-year-old girl was registered at our hospital with the diagnosis of sacrococcygeal tumor stage III. She had undergone surgery, chemotherapy, and radiotherapy. She was referred to department of palliative care when she had second relapse and she was 5 yrs by then.

CT scan showed recurrent disease mass anterior to the coccyx and right para-aortic adenopathy. Palliative chemotherapy was planned for the child.

She came to our department crying with symptoms of pain during daefaecation. Pain was severe in intensity and because of the position of the tumor child was not able to sit. Even when her mother was carrying her, she used to cling to her so that no pressure was felt on sacral area as it would exaggerate her pain.

As the child could not be monitored, on the first day she was started on syrup ibuprofen, xylocaine ointment, and cremaffin syrup. The next day she came back to the department with not much of relief, was admitted and started on oral morphine solution 2.5 mg 4^th^ hrly. Keeping the neuropathic component of pain in mind, syrup carbamazepine 100 mg BD was started. Cremaffin and ibuprofen were continued. By the second day child had good pain relief, was able to lie down on the back and was sleeping comfortably but was constipated. Therefore, lactulose 10 ml at night was started. On the third day, the child was comfortable and had passed stools. On the fourth day, she had burning micturation for which increase in fluid intake was advised and disodium hydrogen citrate was added. By the fifth day, the child was very comfortable, was able to sit on chair, and was busy learning drawing from our pharmacist. She was discharged. One week later, she came back with increase in pain and difficulty in micturation and defaecation. The disease was advancing and the pressure symptoms and pain were increasing. Steroid was added and oral morphine solution was increased to 5 mg/5 ml 4^th^ hrly. At 15 days follow up, morphine was increased to 10 mg/5 ml 4^th^ hrly. She was able to sit comfortably. This was the last we heard about the child. She succumbed to her malignancy.

## DISCUSSION

There is lack of valid epidemiological data on malignancy associated pain in pediatric oncology.[1] The management of pain in palliative care of children is somewhat different from that in adults. It also differs in approach from the management of other types of chronic pain in childhood. Whereas once opioids were thought to be highly dangerous drugs, unsuitable for use in children, they have now taken their place as the mainstay for provision of good analgesia to manage moderate to severe pain in both malignant and non-malignant life limiting conditions. There are relatively little clinical or laboratory data regarding opioids specifically in children. Early research in children does suggest some significant difference in opioid pharmacokinetics particularly with respect to morphine clearance, which seems to be faster in adult.[2]

Thus, the use of opioids in pediatric palliative care presents some unique challenges. Confident and rational use of opioids, illustrated by WHO Guidelines,[3] is essential for adequate management of pain in children with life limiting conditions. In some studies on WHO step 2, tramadol was almost the only opioid used and on WHO step 3, morphine was at least part of regimen.[4]

In our experience, though we treat cancer pain in children, the number of children, under 5 yrs is rather infrequent. We found that there is need for judicious and careful monitoring in titration of oral morphine solution along with adjuvants to achieve good control of pain and thus contribute towards improving quality of life in children with advanced malignancies.
